# ssDNA-PLA, a proximity ligation assay to interrogate DNA damage response proteins involved in homologous recombination

**DOI:** 10.1093/biomethods/bpag003

**Published:** 2026-01-23

**Authors:** Yunhan Yang, Yanping Li, Xiao-Xin Sun, Mu-Shui Dai

**Affiliations:** Department of Molecular and Medical Genetics, School of Medicine, Oregon Health and Science University, 3181 SW Sam Jackson Park Road, Portland, OR 97239, United States; Department of Molecular and Medical Genetics, School of Medicine, Oregon Health and Science University, 3181 SW Sam Jackson Park Road, Portland, OR 97239, United States; Department of Molecular and Medical Genetics, School of Medicine, Oregon Health and Science University, 3181 SW Sam Jackson Park Road, Portland, OR 97239, United States; Department of Molecular and Medical Genetics, School of Medicine, Oregon Health and Science University, 3181 SW Sam Jackson Park Road, Portland, OR 97239, United States; OHSU Knight Cancer Institute, Oregon Health and Science University, 3181 SW Sam Jackson Park Road, Portland, OR 97239, United States

## Abstract

DNA end resection is critical for DNA double-strand break repair via homologous recombination (HR) and replication-coupled repair. Traditional approaches for detecting DNA end resection in cells include fluorescence imaging for replication protein A foci, 5-bromo-2′-deoxyuridine (BrdU) labeling followed by anti-BrdU staining under native conditions to detect ssDNA, and quantitative PCR to detect single-stranded DNA (ssDNA) using the ER-AsiSI U2OS cell system. Here, we comprehensively examined a proximity ligation assay (PLA)-based approach, named ssDNA-PLA, to detect protein-ssDNA interaction by combining BrdU genome-wide DNA labeling with PLA using anti-BrdU and antibody against proteins of interest. We showed that the ssDNA-PLA method is a robust and reliable approach to detect proteins interacting with ssDNA in cells in response to DNA damage induced by various agents and replication stress, including known ssDNA-binding proteins replication protein A, RAD51, and BLM. This approach can be used for studying the proximity of proteins to ssDNA that play roles in DNA end resection and HR repair.

**Keywords** DNA damage repair, DNA end resection, PLA, ssDNA, RPA

## Introduction

DNA double-strand breaks (DSBs) are a severe form of DNA damage that threatens genome stability and impacts normal cellular function [1]. DSBs arise from a variety of endogenous and exogenous sources. Endogenous causes include stalled or collapsed DNA replication forks, replication–transcription collisions, oxidative damage generated by metabolic byproducts such as reactive oxygen species, programmed breaks during V(D)J recombination or meiosis, and strand breaks caused by aberrant topoisomerase activity [2, 3]. Exogenous factors include ionizing radiation, replication stress induced by ultraviolet light, and chemotherapeutic agents that generate DNA breaks or collapse replication forks [2, 4, 5]. If unrepaired, DSBs can lead to chromosomal rearrangements, gene deletions, or cell death, posing a major threat to genomic integrity [6–8]. To maintain genomic stability, cells employ multiple DNA damage repair pathways, with DSBs primarily resolved through nonhomologous end joining (NHEJ) or homologous recombination (HR) [9]. Unlike NHEJ, which directly ligates the broken ends, HR requires a critical step-DNA end resection. In this process, the 5′ end of the break is progressively degraded by nucleases to generate 3′ single-stranded DNA (ssDNA) overhang [10]. DNA end resection not only determines the choice of repair pathway via HR over NHEJ but also plays a crucial role in DNA replication fork stability and genome surveillance mechanisms [9].

Following the generation of ssDNA at stalled replication forks or processed DSBs [11], replication protein A (RPA) rapidly coats the exposed ssDNA to stabilize it and initiate ATR-ATRIP-mediated checkpoint signaling [12]. Additional DNA damage response (DDR) factors, including the BLM helicase, DNA2 and EXO1 nucleases, BRCA1 and BRCA2, associate with ssDNA to mediate long-range end resection, promote RAD51 filament assembly, or protect stalled forks [13–15]. Because of the central role of ssDNA intermediates in these processes, reliable methods for monitoring protein association with ssDNA are essential. Traditional approaches for detecting DNA end resection in cells include fluorescence imaging of endogenous RPA foci or labeling genomic DNA with 5-bromo-2′-deoxyuridine (BrdU), a thymidine analog incorporated into newly synthesized DNA during replication [16], followed by anti-BrdU staining under native (non-denaturing) conditions to detect ssDNA [17, 18], or quantitative PCR-based detection of ssDNA using the ER-AsiSI U2OS system [19, 20]. However, non-denaturing BrdU immunofluorescence reveals ssDNA formation but does not directly measure protein association with these regions [18]. Several additional approaches have been developed, each with distinct strengths and limitations. Chromatin immunoprecipitation can measure protein occupancy on DNA but cannot distinguish protein binding on ssDNA from binding on double-stranded DNA (dsDNA) [21]. Nascent-strand labeling techniques such as iPOND permit protein–DNA complex isolation but require large numbers of cells and label long stretches of chromatin-rich nascent DNA, resulting in low resolution and high background that obscure short ssDNA intermediates and low-abundance repair proteins [22].

BrdU incorporated into intact dsDNA is masked by base pairing and cannot be detected by anti-BrdU antibodies under non-denaturing conditions. Only when DNA undergoes end cleavage and becomes single-stranded is BrdU exposed and specifically recognized by anti-BrdU antibodies [23, 24]. Thus, a proximity ligation assay (PLA) that measures the spatial proximity between BrdU and ssDNA-binding proteins enables sensitive, in situ visualization and quantification of DDR proteins bound to ssDNA. In this study, we systematically examined and optimized the assay across a range of experimental conditions, including different cell systems, alternative anti-BrdU antibodies, various DNA damage inducers, and varied treatment durations. We referred to this assay as the ssDNA-PLA. We further evaluated its applicability to DDR proteins beyond RPA and demonstrated that the ssDNA-PLA assay is a robust and reliable approach for assessing protein–ssDNA interactions in response to diverse DNA damage agents.

## Materials and methods

### Cell lines

Human U2OS and HeLa cells were obtained from ATCC and cultured in Dulbecco’s modified Eagle’s medium supplemented with 10% (v/v) fetal bovine serum (FBS), 50 U/ml penicillin, and 0.1 mg/ml streptomycin at 37°C in a humidified incubator with 5% CO_2_. Cell lines were passaged fewer than 30 times for a maximum of 2 months and routinely monitored for mycoplasma contamination [25].

### Antibodies and reagents

Anti-RPA2 phospho S33 (Abcam, ab211877, PLA 1:1000), Anti-RAD51 (Abcam, ab133534, PLA 1:1000), Anti-BRCA1 (Cell Signaling Technology, IB; 1:1000), Anti-BLM (Invitrogen, PA5-77880; PLA 1:200), Anti-BrdU (Roche, 11170376001, PLA 1:100, primarily used in this study), Anti-BrdU (BD Biosciences, Clone B44, Cat 347580, PLA 1:100, used in Fig. 4C), and anti-tubulin (Proteintech, 66240-1-Ig, IB 1:10000) were purchased. BrdU, etoposide (ETO), camptothecin (CPT), and hydroxyurea (HU) were purchased from Sigma-Aldrich.

### Gene knockdown by RNA interference

RNAi-mediated gene knockdown was performed essentially as previously described [26]. The 21-nucleotide siRNA duplexes with a 3′ dTdT overhang were synthesized by Dharmacon Inc. (Lafayette, CO). The target sequences for BRCA1 were 5′-GGAACCUGUCUCCACAAAG-3′. The control scramble RNA was previously described [27]. These siRNA duplexes (100 nM) were introduced into cells using Lipofectamine 2000 (Invitrogen) following the manufacturer’s protocol. Cells were harvested at 48 hours post-transfection for immunoblot (IB) and PLA analysis.

### Immunoblot

Cells were lysed in NP40 lysis buffer consisting of 50 mM Tris–HCl (pH 8.0), 0.5% nonidet P-40, 1 mM EDTA, 150 mM NaCl, 1 mM phenylmethylsulfonyl fluoride, 1 mM dithiothreitol, 1 μg/ml pepstatin A, and 1 mM leupeptin with brief sonication. Equal amounts of total protein were used for IB analysis.

### BrdU incorporation, DNA damage treatment. and cell preparation

Cells were seeded on glass coverslips in 24-well plates at a density of 3–4 × 10^4^ cells per well and incubated with 10 μM BrdU for 20 hours to allow incorporation into newly synthesized DNA. The cells were then treated with different DNA-damaging agents ETO (20 µM), CPT (2 µM), X-ray (10 Gy), for 2 hours, or HU (10 mM) for 4 hours. Cells were washed with PBS and pre-extracted on ice for 10 minutes in buffer A (10 mM PIPES pH 7.0, 100 mM NaCl, 3 mM MgCl_2_, 1 mM EGTA, 0.5% Triton X-100, 300 mM sucrose), followed by incubation in cytoskeleton stripping buffer B (10 mM Tris pH 7.5, 10 mM NaCl, 3 mM MgCl_2_, 1% Tween-20, 0.5% sodium deoxycholate) for 10 minutes. After washing in PBS, cells were fixed with 4% paraformaldehyde for 15 minutes and post-fixed in cold 100% methanol at -20°C for 5 minutes. Permeabilization was performed with 0.5% Triton X-100 for 15 minutes at room temperature, followed by blocking in 3% BSA.

### BrdU-based PLA assay

**Figure 1:**
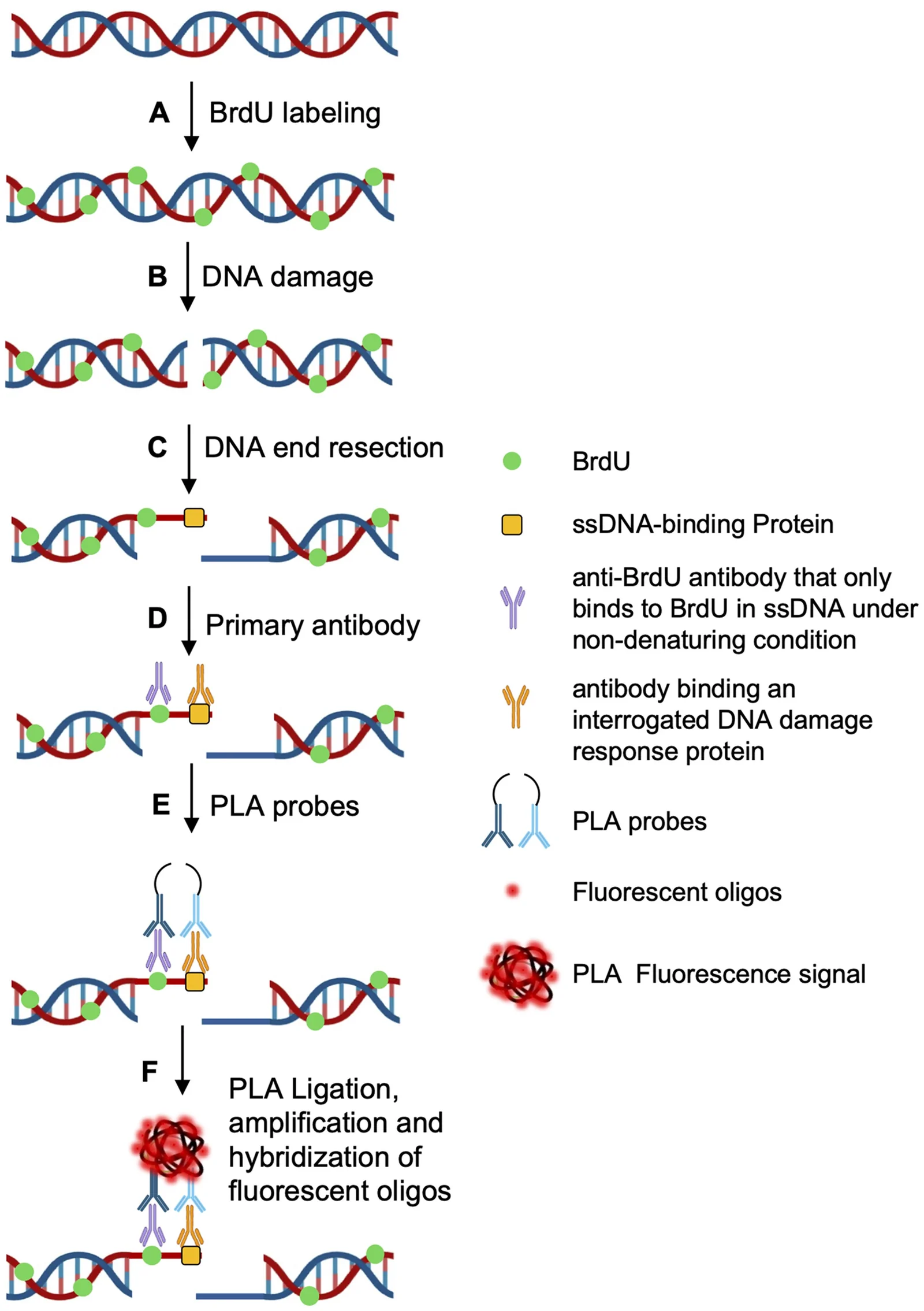
Schematic overview of BrdU-based proximity ligation assay for detection of protein–ssDNA interaction. Cells cultured in the presence of 10 uM BrdU (A) are subjected to induction of DSBs (B). DNA end resection (C) at damaged sites results in the generation of ssDNAs. After fixation, the cells are incubated with primary antibody against ssDNA-binding protein of intertest (used in this study: anti-RPA2 phospho S33 rabbit polyclonal antibody, Abcam, ab211877; anti-RAD51 rabbit monoclonal antibody, Abcam, ab133534; anti-BLM rabbit polyclonal antibody, Invitrogen, PA5-77880) and anti-BrdU mouse monoclonal antibody (Roche, 11170376001 or BD Biosciences, 347580) that only binds to BrdU in ssDNA under non-denaturing conditions (D), followed by incubation with secondary antibody coupled with oligonucleotides (Duolink^®^ In Situ PLA^®^ anti-mouse MINUS and anti-rabbit PLUS probes (Sigma-Aldrich) (E). When the ssDNA-binding protein is in close with BrdU, the connector oligonucleotides join the PLA probes and mediate ligation to form a circular DNA template. The resulting circular DNA template is then amplified by rolling circle amplification, followed by hybridization of fluorescently labeled detection oligonucleotides, producing discrete fluorescent spots/signals (F)

PLA detection of BrdU-associated proteins was carried out using the Duolink In Situ Red Fluorescence Kit (Sigma-Aldrich) with minor adjustments to the manufacturer’s protocol and is summarized in Fig. 1. Fixed and blocked samples were incubated with primary antibodies against BrdU and ssDNA-binding proteins (RPA2, RAD51, or BLM) overnight at 4°C. Slides were then washed with Duolink Wash Buffer A and subsequently incubated with PLA probes (PLUS and MINUS, 1:5 dilution) for 1 hour at 37°C. After washing, the ligation reaction was carried out by incubating slides in ligase diluted 1:40 in 1× ligation buffer for 30 minutes at 37°C. After washing with Buffer A, rolling-circle amplification was subsequently conducted by incubating samples with polymerase diluted 1:80 in 1× amplification buffer at 37°C in a pre-heated humidified chamber. Slides were then washed with Wash Buffer B, briefly rinsed in 0.01× Wash Buffer B, and air-dried. Finally, samples were mounted using Duolink^®^ In Situ Mounting Medium containing DAPI, covered with coverslips. Samples were equilibrated for 15 minutes before imaging.

### Imaging and statistical analyses

Confocal imaging was carried out using a Zeiss LSM980 laser-scanning microscope equipped with a Plan-Apochromat 63×/1.40 NA oil-immersion objective. Fluorescent signals were acquired using the following excitation and emission settings: DAPI, 405-nm excitation and 420–480-nm emission; Alexa Fluor 555, 561-nm excitation with 565–620-nm detection. PLA puncta were quantified using ImageJ as the number of discrete nuclear foci per cell. For each PLA condition, at least 100 cells were examined. Data are reported as mean ± SD. Statistical analyses were conducted in GraphPad Prism. Comparisons between two conditions were evaluated using an unpaired, two-sided Student’s t-test. For experiments involving more than two groups, two-way ANOVA followed by Bonferroni post-hoc testing was applied. Statistical significance was defined as *P* < .05.

## Results

### Detection of RPA binding to ssDNA by BrdU-RPA PLA in cells

**Figure 2:**
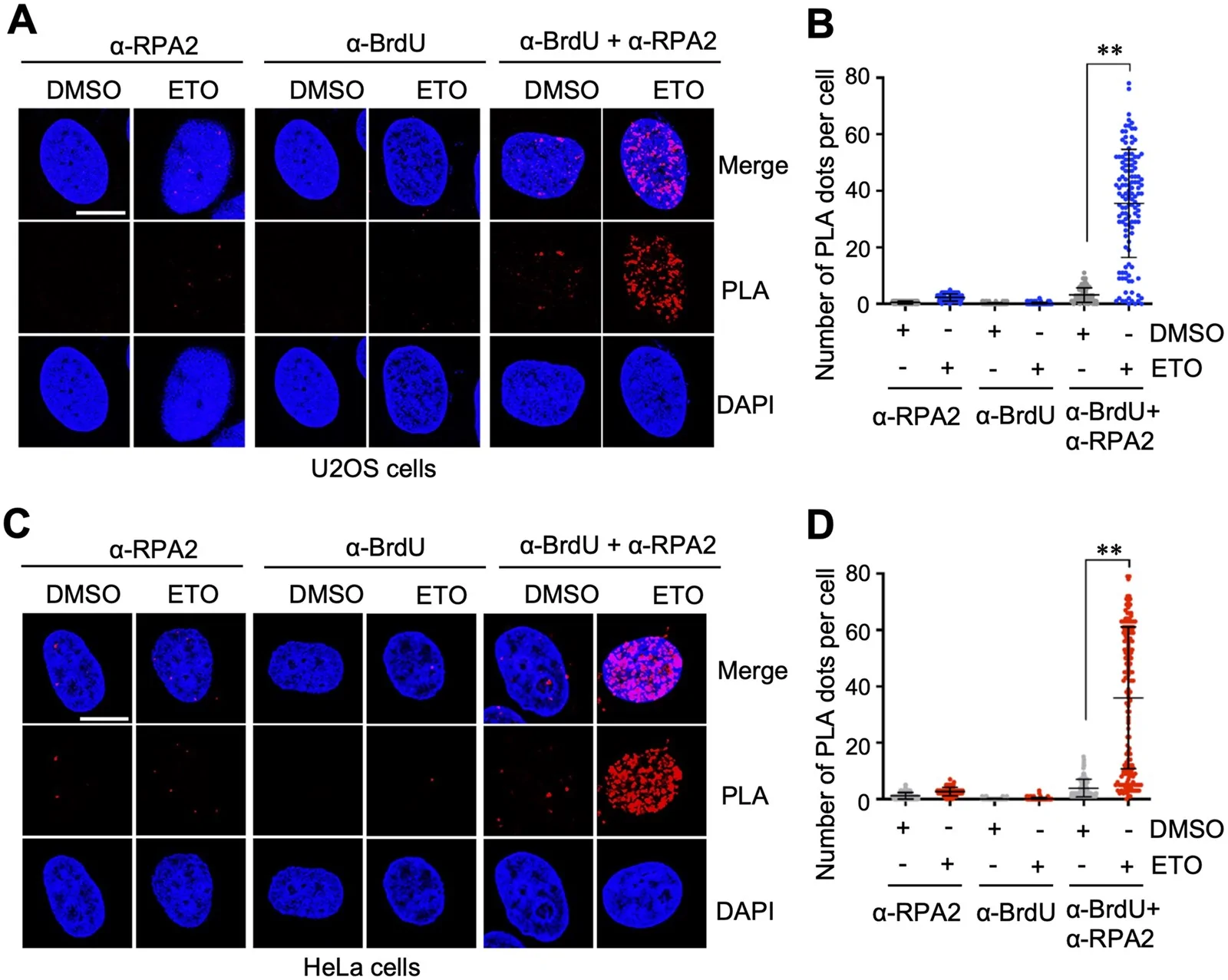
RPA2–ssDNA interaction detected by anti-BrdU and anti-RPA2 PLA upon DNA damage. U2OS (A, B) and HeLa (C, D) cells cultured in the presence of 10 uM BrdU for 20 hours were treated with DMSO or 20 uM ETO for 2 hours. The cells were assayed by PLA using anti-BrdU (Roche) and anti-RPA2 (phosphor S33) antibodies. Shown are representative confocal images (A, C) and the quantification (B, D). Scale bar, 10 μm. **P < .01, compared to DMSO-treated control group

RPA coats ssDNA after DNA end resection in response to DSBs [12]. To measure RPA binding to ssDNA, we employed a BrdU labeling and PLA method. We first performed the assay in U2OS cells. Cells were pre-labeled with BrdU and subsequently treated with ETO to induce DSBs. The cells were then assayed by PLA using anti-BrdU and anti-phospho-RPA2 antibodies in cells under native conditions. Compared with DMSO-treated controls, ETO treatment markedly increased the number of BrdU-RPA2 PLA signals per nucleus, indicating RPA2 binding to ssDNA and active DNA end resection (Fig. 2A and B; Supplementary Fig. S1A). As negative controls, staining with anti-phospho-RPA2 or anti-BrdU antibody alone did not give rise to significant PLA signals (Fig. 2A and B), indicating that the PLA signals are specific for RPA2 association with BrdU incorporated ssDNAs. Similar results were also observed in HeLa cells (Fig. 2C and D; Supplementary Fig. S1B). These results confirm the robustness and cross-cell-line applicability of the BrdU-RPA2 PLA method.

### Detection of ssDNA binding by BLM and RAD51 using ssDNA-PLA assays

**Figure 3:**
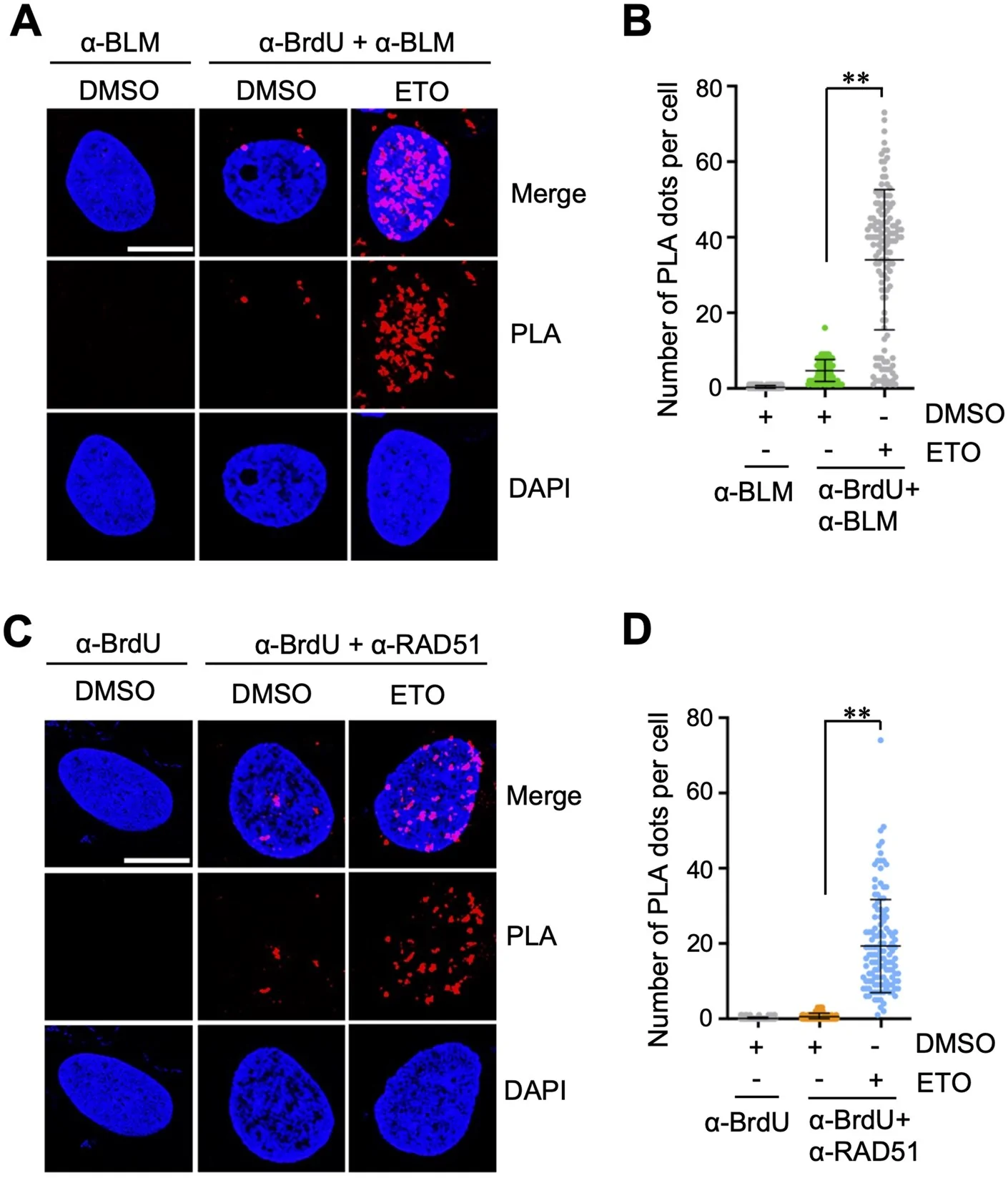
Detection of the binding of BLM and RAD51 to ssDNA upon DNA damage. U2OS cells cultured in the presence of 10 uM BrdU for 20 hours were treated with DMSO or 20 uM ETO for 2 hours and assayed by PLA using anti-BrdU and anti-BLM antibodies (A, B) or anti-BrdU and anti-RAD51 antibodies (C, D). Shown are representative confocal images (A, C) and the quantification (B, D). Scale bar, 10 μm. **P < .01, compared to DMSO-treated control group

Several proteins critical for DDR and DNA damage repair interact with ssDNA following DNA end resection, including the BLM helicase [28, 29] and the recombinase RAD51 [30, 31]. To extend the utility of this assay beyond RPA2, we next examined whether ssDNA binding by BLM and RAD51 can be detected by the ssDNA-PLA methods. Indeed, both BrdU-BLM (Fig. 3A and B; Supplementary Fig. S2A) and BrdU-RAD51 (Fig. 3C and D; Supplementary Fig. S2B) PLA signals were significantly increased in cells treated with ETO compared to DMSO-treated cells. These results demonstrate that the BrdU-based PLA strategy can be generally adapted to monitor interactions between ssDNA and various ssDNA-binding DNA repair factors.

### Analysis of RPA-ssDNA binding upon treatment with various genotoxic agents

**Figure 4:**
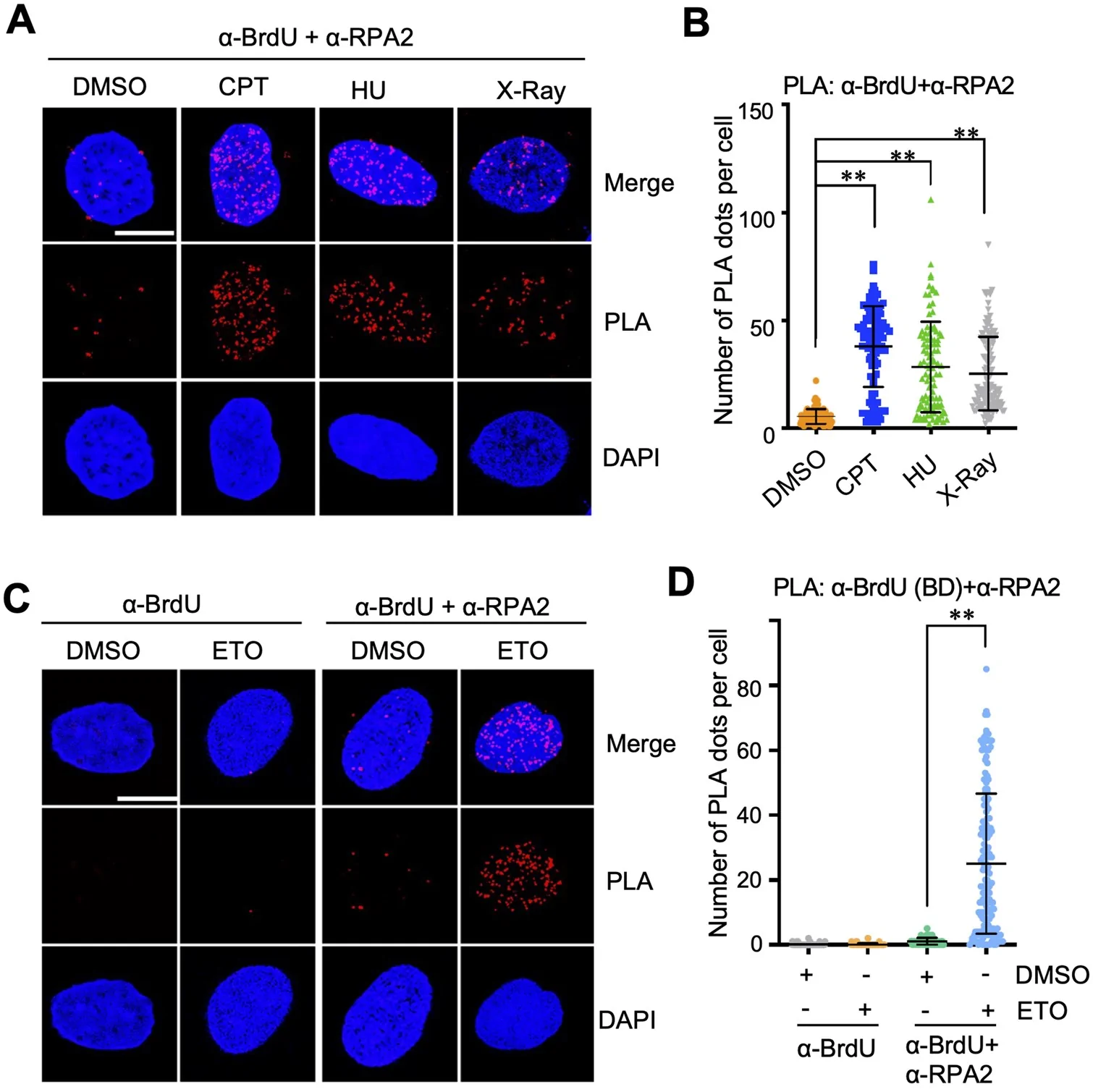
Analysis of DNA damage-induced resection upon treatment with various genotoxic agents. (A, B). U2OS cells were cultured in the presence of 10 uM BrdU for 20 hours and then treated with 2 µM CPT or 10 GY X-ray for 2 hours or 10 mM HU for 4 hours. The cells were assayed by PLA using anti-BrdU and anti-RPA2 (phosphor S33) antibodies. (C, D). U2OS cells cultured in the presence of 10 uM BrdU for 20 hours were treated with DMSO or 20 uM ETO for 2 hours and assayed by PLA using a second anti-BrdU antibody (BD Bioscience) and anti-RPA2 (phosphor S33) (C, D). Shown are representative confocal images (A, C) and the quantification (B, D). Scale bar, 10 μm. **P < .01, compared to DMSO-treated control group

To test whether different types of DNA lesions induce distinct resection responses, ssDNA-PLA assays were performed in U2OS cells treated with additional DNA-damaging agents, including CPT, HU, and X-Ray irradiation. As shown in Fig. 4A and B, and Supplementary Fig. S3A, BrdU-RPA2 PLA signals were all markedly increased in cells treated with these agents. These data further demonstrate the application of the ssDNA-PLA method for detecting protein–ssDNA interaction in cells in response to various sources of replication stress and DSBs.

### Analysis of RPA-ssDNA PLA using an alternative anti-BrdU antibody

To assess whether the choice of BrdU antibody affects the detection of DNA end resection, we performed BrdU-RPA2 PLA assays using a different anti-BrdU antibody. We observed a similar increase in BrdU-RPA2 PLA signals in cells treated with ETO compared with DMSO-treated controls (Fig. 4C and D; Supplementary Fig. S3B). These results indicate that the assay is robust and that different BrdU antibodies yield consistent results.

### Time course analysis of DNA end resection after ETO treatment and drug removal

**Figure 5:**
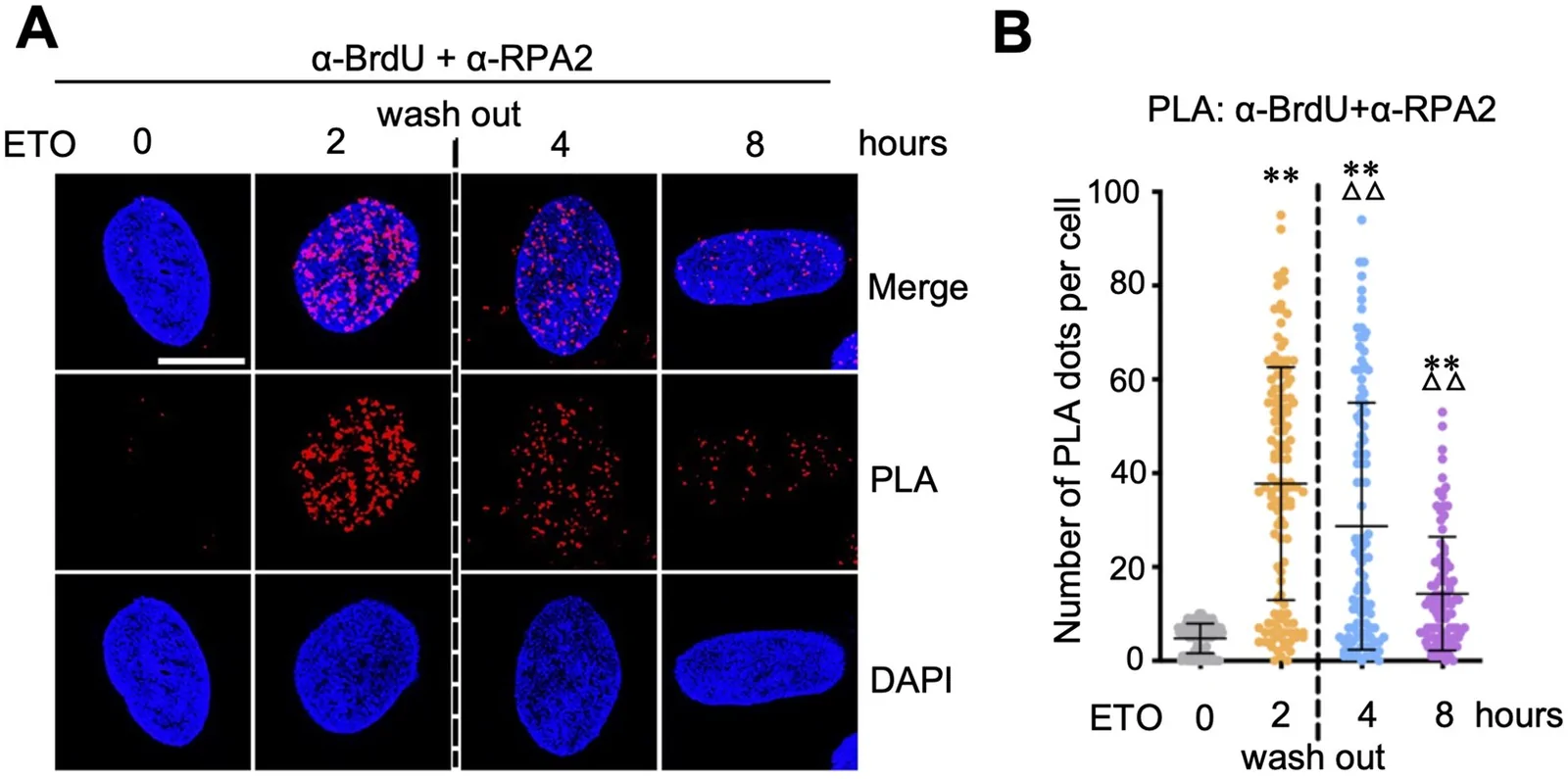
Monitoring the dynamics of DNA end resection and DNA repair by time course analysis of RPA-ssDNA PLA. U2OS cells were cultured in the presence of 10 uM BrdU for 20 hours followed by treatment with 20 uM ETO. The cells were washed after 2 hours of ETO treatment with the replacement of fresh culture medium. The cells were fixed at different time points and assayed by PLA using anti-BrdU and anti-RPA2 (phosphor S33) antibodies. Shown are representative confocal images (A) and the quantification (B). Scale bar, 10 μm. **P < .01, compared to untreated (0 hour) control group. ^△△^*P* < .01, compared to 2 hour ETO treatment group

We next assessed the ssDNA-PLA approach to observe the dynamics of DNA end resection. U2OS cells treated with ETO for 2 hours were subjected to washing to remove the remaining drug followed by chasing RPA–ssDNA interaction over different time points. As shown in Fig. 5A and B, and Supplementary Fig. S4, a pronounced increase in BrdU-RPA2 PLA signals was observed at 2 hours post-ETO treatment, consistent with rapid initiation of end resection in response to ETO-induced DSBs. Following drug removal, BrdU-RPA PLA signals progressively declined over time, with a clear reduction by 4 hours and further loss by 8 hours post-treatment, indicating that DNA end resection is an early and transient response to DSB induction. These time-course data demonstrate that the PLA assay is suitable for monitoring dynamic changes in DNA end resection and protein–ssDNA binding.

### ssDNA-PLA assay upon knockdown of DNA end resection regulator BRCA1

**Figure 6:**
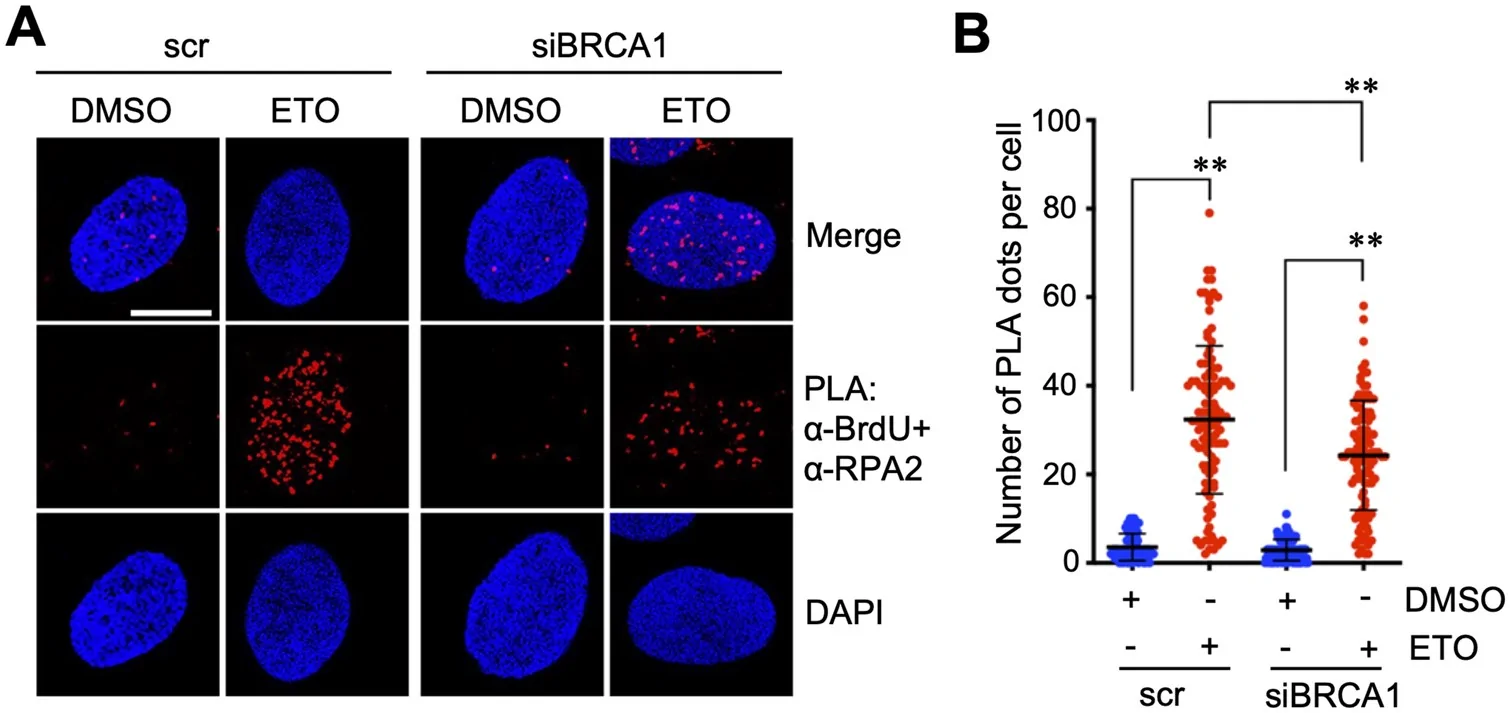
Investigation of BRCA1 knockdown-mediated inhibition of DNA end resection by ssDNA-PLA. U2OS cells transfected with scrambled (scr) or BRCA1 siRNA were cultured in the presence of 10 uM BrdU for 20 hours followed by treatment with 20 uM ETO for 2 hours. The cells were assayed by PLA using anti-BrdU and anti-RPA (phosphor S33) antibodies. Shown are representative confocal images (A) and the quantification (B). Scale bar, 10 μm. **P < .01, compared to control group

To further investigate the application of ssDNA-PLA in the regulation of DNA end resection during DDR, we sought to examine whether we can detect the changes in DNA end resection upon depletion of BRCA1, which plays a key role in DNA end resection [32, 33]. U2OS cells transfected with scrambled control or BRCA1 siRNA were subjected to BrdU labeling and ETO treatment followed by BrdU-RPA2 PLA assays. We observed that ETO treatment-induced increase of BrdU-RPA2 PLA signals per nucleus was significantly reduced in cells transfected with BRCA1 siRNA compared to scrambled RNA-transfected cells (Fig. 6A and B; Supplementary Fig. S5A), consistent with BRCA1’s critical function in DNA end resection [32, 33]. BRCA1 protein was efficiently knocked down by BRCA1 siRNA as determined by IB analysis (Supplementary Fig. S5B). These results demonstrate that the ssDNA-PLA assay is sensitive to perturbations in key resection factors such as BRCA1 and can be used to evaluate genetic regulation of DNA end resection.

## Discussion

In this study, we describe an ssDNA-PLA method to detect protein–ssDNA interactions in cells. The method is based on BrdU labeling of genomic DNA and PLA detection of protein–ssDNA interactions using anti-BrdU antibody and antibodies against proteins of interest under non-denaturing conditions. We demonstrate that the assay can robustly detect the binding of RPA2, BLM, and RAD51 to ssDNA following DNA DSBs induced by diverse DNA-damaging agents in situ with single-cell resolution. Additionally, we showed that the assay can be used to monitor DNA end resection and to investigate the function of proteins involved in DNA end resection and DNA damage repair, as exemplified by BRCA1.

Fowler and Tyler have previously described the PLA-based assay to detect RPA–ssDNA interaction in U2OS cells [34]. In their study, the cells were cultured with BrdU for 48 h and then re-seeded in culture for an additional 18–24 hours before DNA damage treatment. We shortened BrdU incubation time and showed that 20 hours of culture with BrdU is enough for the detection of protein-ssDNA PLA signals, although only one strand of each replicated DNA molecules incorporated BrdU during this time (see Fig. 1). This is supported by a PLA detection of BrdU-CtIP interaction by Wang et al. [35] in which a 24-hour BrdU incubation was used. We applied this approach to detect the interactions of multiple DDR proteins with ssDNA and in additional cell lines. We also tested different anti-BrdU antibodies that gave rise to reproducible results. Together, these data demonstrate that the ssDNA-PLA assay is highly useful for detecting the protein–ssDNA interactions and, accordingly, measuring DNA end resection, which is more specific than indirect staining and visualization of the resected DNA by RPA or BrdU fluorescence foci. Conceivably, this approach can be extended to study protein–ssDNA interaction in other cellular processes such as DNA replication.

The assay is straightforward, and as with any other assay, proper controls, including staining with anti-BrdU alone or an antibody against the protein of interest, are needed to ensure specific detection of PLA signals. The BrdU incorporation time to ideally label all cells with BrdU may vary depending on how quickly the cells proliferate. A 20–24-hour incubation is typically sufficient for most highly proliferative cancer cell lines with a 20–24 doubling time. The incubation time should be adjusted for cells with longer doubling times. Continuous BrdU incubation does not affect cell viability but may alter cell cycle progression [36]. Primary antibody dilutions should be optimized in preliminary tests to achieve clear signal-to-background discrimination and should be kept constant across all experiments. Because the PLA assay permits detection of two target molecules in close proximity (within approximately 40 nm), proteins bound to dsDNA at the dsDNA/ssDNA junctions could be detected. The method would also detect the proteins of interest that indirectly interact with-ssDNA via binding to other DDR proteins involved in the processing of resected ends. Thus, the ssDNA-PLA assay remains a valuable tool for studying protein function in HR repair and DNA replication.

## Supplementary Material

bpag003_Supplementary_Data

## Data Availability

All data generated or analyzed in this study are included in this article and its Supplementary Information files.
