# Supplementary material for: ssDNA-PLA, a proximity ligation assay to interrogate DNA damage response proteins involved in homologous recombination
Source: Biol Methods Protoc. 2026 Jan 23;11(1):bpag003. doi: 10.1093/biomethods/bpag003 (PMC12910372; doi:10.1093/biomethods/bpag003)
Supplement: bpag003_Supplementary_Data [file bpag003_supplementary_data.pdf]

Supplementary Information

ssDNA-PLA, a proximity ligation assay- based approach to detect protein-single strand DNA interaction and DNA end resection in cells

Yunhan Yang[1], Yanping Li[1], Xiao-Xin Sun[1]*, Mu-Shui Dai[1,2]*

[1]Departments of Molecular & Medical Genetics, School of Medicine, [2]the OHSU Knight Cancer Institute, Oregon Health & Science University, 3181 SW Sam Jackson Park Road, Portland, OR 97239, USA

This PDF file includes:

Supplementary Figures 1 to 5

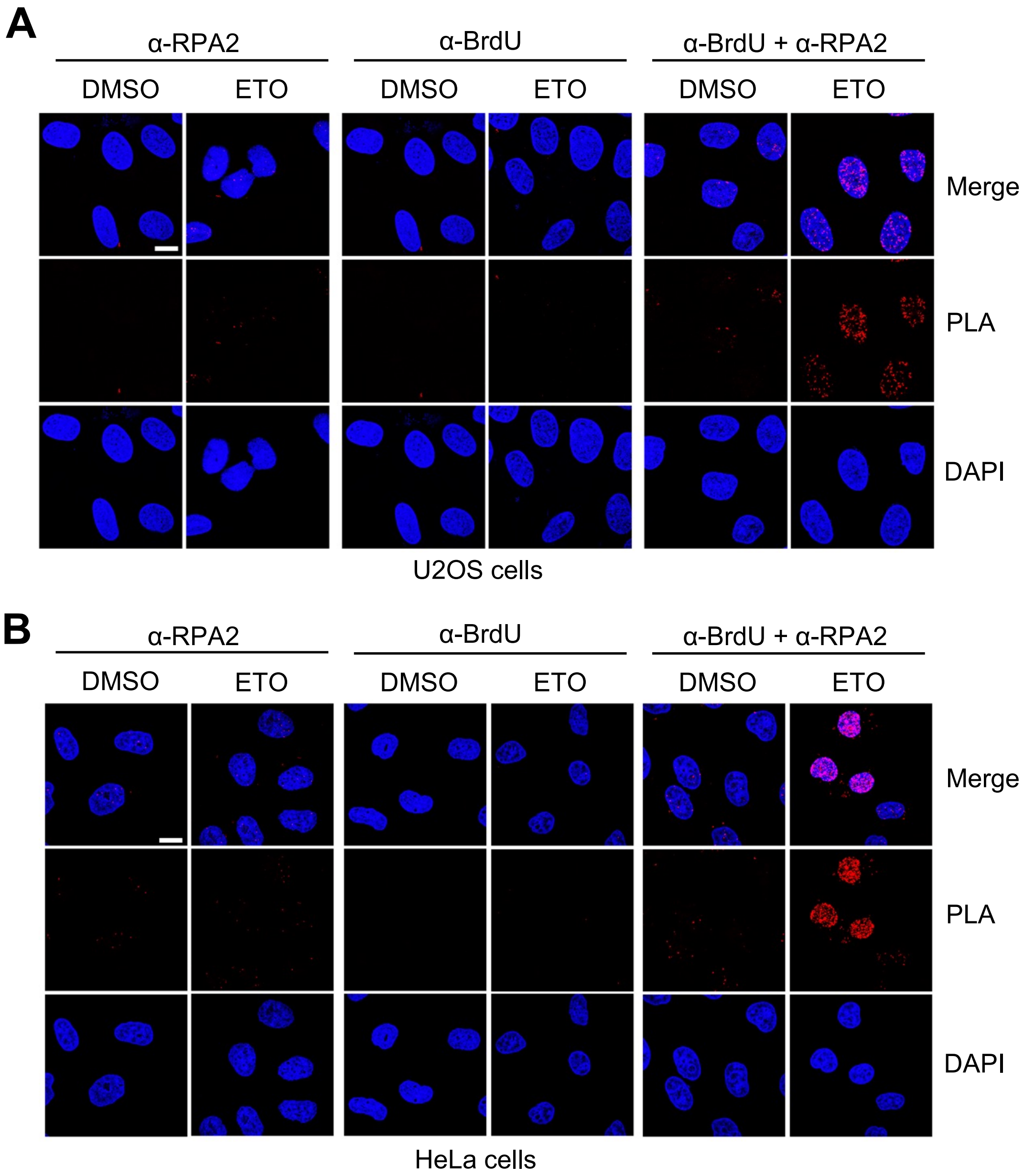

Figure S1. RPA-ssDNA interaction detected by anti-BrdU and anti-RPA PLA upon DNA damage. U2OS (A) and HeLa (B) cells cultured in the presence of 10 uM BrdU for 20 hours were treated with DMSO or 20 uM ETO for 2 hours. The cells were assayed by PLA using anti-BrdU and anti-RPA antibodies. Shown are representative confocal images. Scale bar, 10 μm.

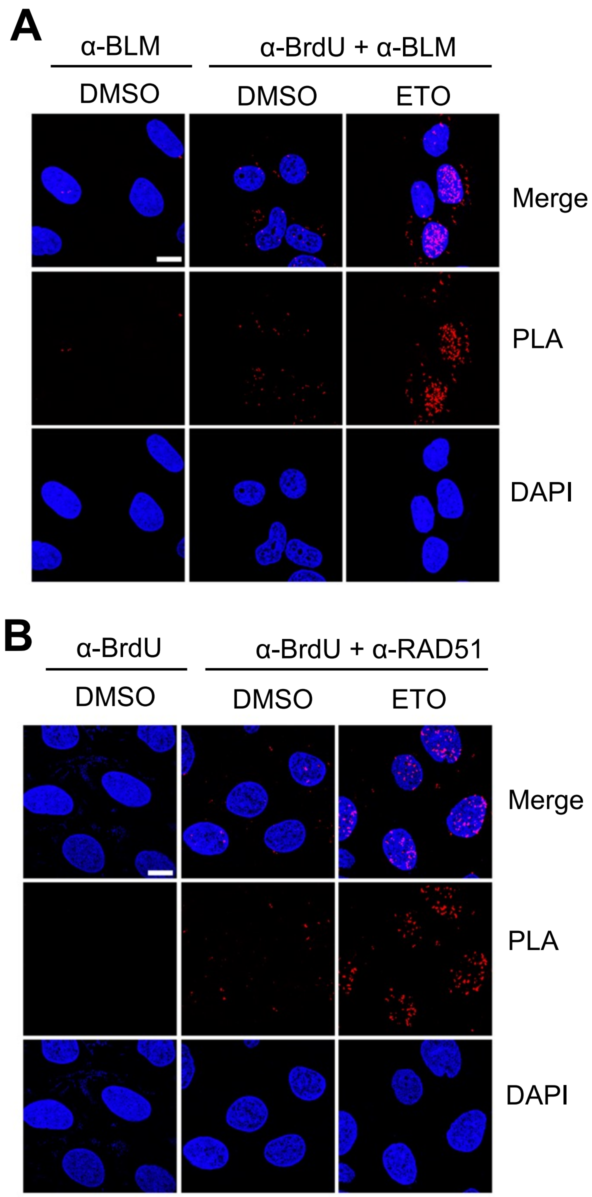

Figure S2. Detection of the binding of BLM and RAD51 with ssDNA upon DNA damage. U2OS cells were cultured in the presence of 10 uM BrdU for 20 hours and then treated with DMSO or 20 uM ETO for 2 hours. The cells were assayed by PLA using anti-BrdU and anti-BLM antibodies (A) or anti-BrdU and anti-RAD51 antibodies (B). Shown are representative confocal images. Scale bar, 10 μm.

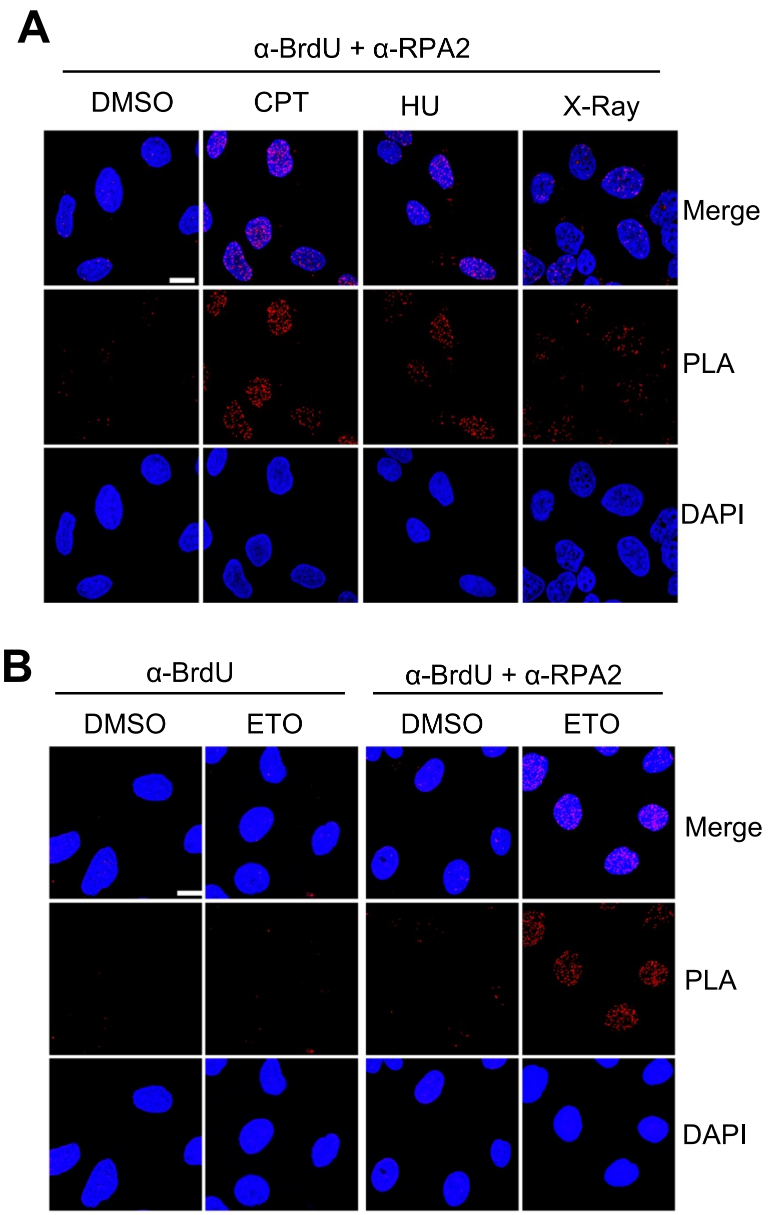

Figure S3. Analysis of RPA-ssDNA binding in cells upon treatment with various genotoxic agents by ssDNA-PLA. **(A)** U2OS cells were cultured in the presence of 10 uM BrdU for 20 hours and then treated with 2 μM CPT or 10 GY X-ray for 2 hours or 10 mM HU for 4 hours. The cells were assayed by PLA using anti-BrdU and anti-RPA2(phosphor S33) antibodies. (**B**). U2OS cells cultured in the presence of 10 uM BrdU for 20 hours were treated with DMSO or 20 uM ETO for 2 hours and assayed by PLA using a second anti-BrdU antibody (BD Bioscience) and anti-RPA2 (phosphor S33). Shown are representative confocal images. Scale bar, 10 μm.

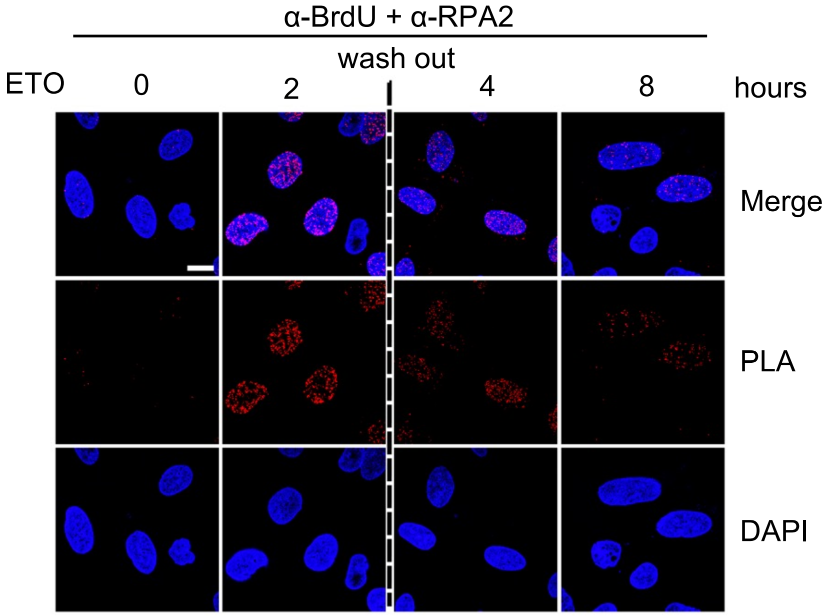

Figure S4. Monitoring the dynamics of DNA end resection and DNA repair by time course analysis of RPA-ssDNA PLA. U2OS cells were cultured in the presence of 10 uM BrdU for 20 hours followed by treatment with 20 uM ETO. The cells were washed after 2 hours of ETO treatment with the replacement of fresh culture medium. The cells were fixed at different time points and assayed by PLA using anti-BrdU and anti-RPA (phosphor S33) antibodies. Shown are representative confocal images. Scale bar, 10 μm.

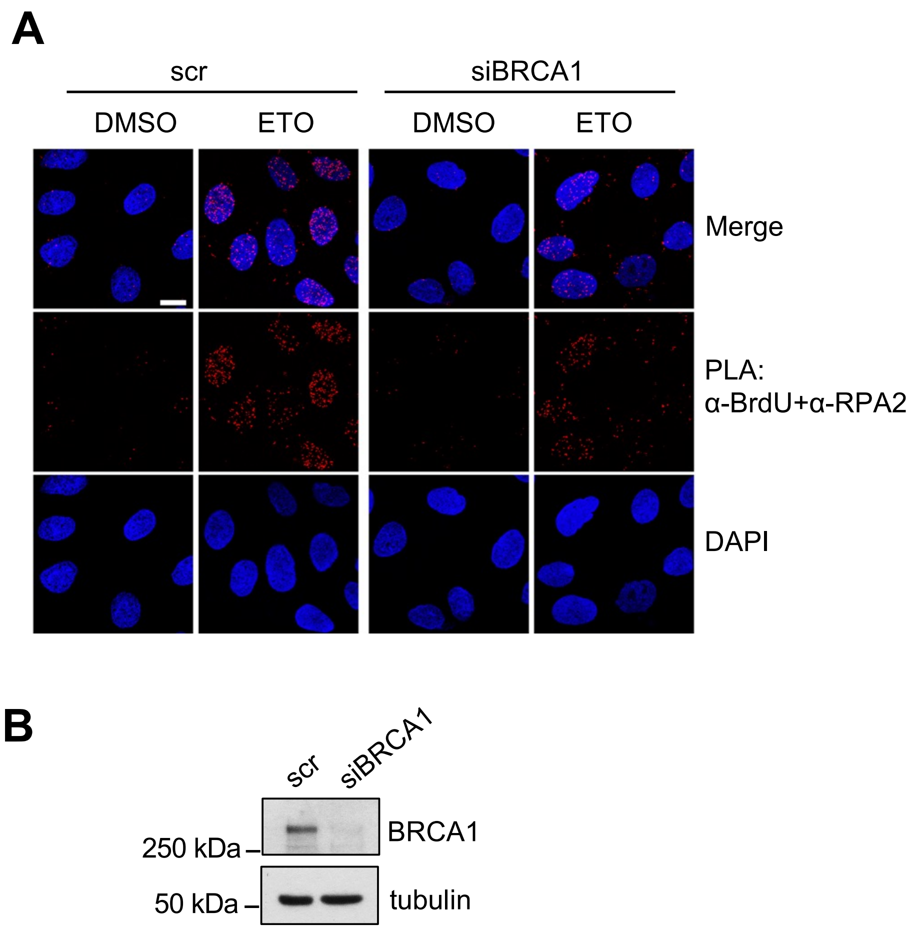

Figure S5. Investigation of BRCA1 knockdown-mediated inhibition of DNA end resection by ssDNA-PLA. U2OS cells transfected with scrambled (scr) or BRCA1 siRNA were cultured in the presence of 10 uM BrdU for 20 hours followed by treatment with 20 uM ETO for 2 hours. The cells were assayed by PLA using anti-BrdU and anti-RPA (phosphor S33) antibodies (A) and by IB detection of BRCA1 protein levels using anti-BRCA1 antibody (B). Shown are representative confocal images (A). Scale bar, 10 μm.
